# Efficacy of a combined protocol for re-insemination of open cows after early pregnancy diagnosis using ultrasonography and its effect on fertility

**Published:** 2013-04-28

**Authors:** A.O. Gaja, S.Y.A. Al-Dahash, C. Kubota, T. Kojima, I. Hatazoe

**Affiliations:** 1 *Department of Surgery and Theriogenology, Faculty of Veterinary Medicine, University of Tripoli, Tripoli, Libya*; 2 *Laboratory of Theriogenology, Department of Veterinary Medicine, Faculty of Agriculture, Kagoshima University, Kagoshima city, Kagoshima province, Japan*; 3 *Kagoshima Agriculture Cooperative Associations, Kagoshima city, Kagoshima province, Japan*

**Keywords:** Early pregnancy diagnosis (EPD), estrus detection, GnRH, Re-insemination protocol, Ultrasonography

## Abstract

The objective of the present field study was to establish the beneficial effects of re-insemination of non-pregnant cows using ultrasonography 20 to 23 days after the artificial insemination. A total of 245 Japanese Black cows were artificially inseminated and early pregnancy diagnosis (EPD) was performed on 92 cows 20 days after insemination, using ultrasonography. The remaining 153 cows were considered as negative controls in which routine rectal palpation was performed for pregnancy diagnosis 45-50 days post-insemination. EPD revealed that eleven of the 92 cows (12%) were infertile due to ovarian abnormalities and were thus excluded from the rest of the study. Forty-eight (59%) of the remaining 81 cows were diagnosed as pregnant, while the other 33 (41%) were diagnosed as non-pregnant. Of these non-pregnant cows, 17 of them received a dose of an analogue of the gonadotropin-releasing hormone (GnRH analogue) and were then timed-inseminated, while the other 16 were observed for estrus signs, and 13 of them (81%) were artificially inseminated. Rates of conception were 35% and 38% in the GnRH and the artificially inseminated groups, respectively (*P*>0.05). Total pregnancy rate for the EPD group increased significantly (74%) (*P*<0.01) when compared to the control cows (54%) within the same period. In conclusion, our field study demonstrated that re-insemination of non-pregnant cows following EPD is highly efficacious not only in improving the rate of fertility via reducing inter-insemination and inter-calving intervals, but also aids in the early detection of ovarian disorders.

## Introduction

On modern cattle farms, any delay in the conception of non-pregnant cows is deemed unacceptable financial loss (Oltenacu *et al.*, 1990). Several methods of early pregnancy diagnosis (EPD) have been employed to detect the non-pregnant cows for the purpose of re-insemination. Such methods include rectal palpation, starting from day 35 post insemination (Thompson *et al.*, 1995), hormonal assay using milk, blood, or feces to detect the progesterone levels (Katagiri *et al.*, 2002, Faustini *et al.*, 2007; Isobe *et al.*, 2005), ultrasonography to detect the embryo(s) starting from day 26 (Ramano *et al.*, 2006) and some other laboratory methods; such as using radioimmunoassay of a pregnancy-specific protein in the plasma and early conception factor (ECF) test (Humblot *et al.*, 1988; Cordoba *et al.*, 2001) or simply the non-return to estrus method.

However, all these methods have few disadvantages regarding the days at which diagnosis is performed; this is because with those methods, the non-pregnant cows cannot be diagnosed early enough to be treated for infertility if they do not exhibit the typical signs of estrus. In such cases they should be synchronized for estrus as soon as possible (Lucy, 2005) or just left to wait until the next natural estrus; which implies wasting of valuable resources such as time, money, and effort (Oltenacu *et al.*, 1990).

Therefore, early detection of non-pregnant cows after insemination is considered an essential process that enables us to make a right decision at a proper time, especially when the early detection method is combined with the re-insemination program. Thus, the objective of the present field study was to establish the advantages of re-insemination of non-pregnant cows diagnosed using ultrasonography as early as 20 to 23 days post insemination.

## Materials and Methods

### Animals

Two hundred and forty five (245) Japanese Black cows (raised in a cattle farm in Sendai city/Japan) were used for this study. Feeding of animals in this farm was according to the standard Japanese Animal Feeding Programs, AFFRC (2004).

### Methods for synchronization of estrus and Insemination

All the cows (n=245) received two doses of intramuscular injections of 500 µg prostaglandin F_2_α analogues (PGF_2_α) (RESIPRON-C, Teikoku Zoki Co. Ltd, Japan) 11 days apart, after at least day 40 postpartum. Artificial insemination by the a.m.- p.m. rule (Nebel *et al.*, 1994) was performed on day’s 4-6 post injection of the second dose of PGF_2_α, when the cows exhibited signs of estrus.

### Method of Pregnancy Diagnosis

EPD was performed according to the procedure described by Gaja *et al*. (2009), which involves monitoring the cross sectional areas of corpora lutea (CLc-s area). Ovaries of 92 cows were scanned using real-time ultrasonography (Tringa linear, Esaotepie medical, Netherlands), equipped with a 5 MH linear transducer. Open (non-pregnant) cows were randomly divided into two groups:

***Group1*** (n=17): Cows received an analogue of the gonadotropin-releasing hormone (GnRH analogue) (10 μg, Buserelin, Teikoku Zoki Co. Ltd, Japan) and were inseminated artificially, using timed artificial insemination (TAI) technique 16-20 hours post GnRH injection. (N.B.: At the time of injection, all the cows in this group had dominant follicles, on their ovaries, of more than 10 mm diameter).

***Group2*** (n=16): Cows were inseminated artificially according to the a.m.-p.m. rule after the detection of estrus.

Ultrasonography scanning was performed by the same expert person; avoiding an invasive manipulation of genitalia. The cross-sectional areas (mm^2^) of CLs (CL c-s areas) were calculated using the following formula:

CL c-s area (elliptical area) = π× (diameter a/2) × (diameter b/2)

{Where (a) and (b) are the long and the short diameter of the CL, respectively}.

Regression rate of the CL was calculated using the following formula:

100- (day X^1^ CL c-s area/ day X^2^ CL c-s area) × 100.

{Where X^1^ and X^2^ represent the 1^st^ and 2^nd^ scanning days, respectively}.

The remaining 153 cows were diagnosed for pregnancy using the routine farm practice (rectal palpation on day’s 45 - 50 post insemination).

### Statistical Analysis

For statistical analysis, significant differences between the pregnant and non-pregnant cows were determined using a chi-square test. A *P*-value less than 0.05 were considered statistically significant.

## Results

All cows (n=245) exhibited estrus and were inseminated artificially. 153 cows were considered as negative controls, in which routine rectal palpation was performed for pregnancy diagnosis 45-50 days post-insemination. EPD was performed on 92 cows 20 days after insemination, using ultrasonography. During the first ultrasonographic examination, ovarian disorders were discovered in 11 cows (12%). These cows were excluded from the remaining part of the experiment. On the second ultrasonographic examination of the remaining 81 apparently-healthy cows, which was performed within days 20 to 23, the results showed that48 (59%) of the remaining 81 cows were diagnosed as positively pregnant. On the other hand, 33 (41%) of the remaining 81 cows were diagnosed as non-pregnant.

The 33 non-pregnant cows were further divided into two groups designated as group 1 and group 2. All cows in group1 (n=17) received GnRH analogue and were inseminated using TAI 16-20 hours post GnRH injection. All cows in group 2 (n=16) were observed for estrus, and 13 of them (81%) were artificially inseminated (AI). Conception rate (which denotes the number of positive pregnancies) was 35% (6 out of 17) in the GnRH +T AI group (group 1) and 38% (6 out of 16) in AI group (group 2), (*P*>0.05).

Total pregnancy rate (1^st^ and 2^nd^ inseminations of treated cows), increased (60/81, 74%) when compared to the pregnancy rate in the 1^st^ inseminated cows of treatment (48/81, 59%) and the cows in the control group (82/153, 54%) within the same period (*P*<0.01).

## Discussion

This study is a field application of our previous research on EPD in Japanese Black cows (Gaja *et al.*, 2009; Gaja *et al.*, 2012), in which we found that encouraging results can be obtained without any delay using transrectal ultrasonographic imaging of corpora lutea cross-sectional areas (CLs c-s areas), a protocol which is followed by double verification on days 13 to 16 and 20 to 23 post insemination. Experimental procedures employed in the present study gave us a unique opportunity for an early diagnosis of pregnancy, especially for the non-conceived cows. Thus, these cows could be re-inseminated as early as possible after being diagnosed as non-pregnant without any undue wasting of more open-days.

In our recent study, (Gaja *et al.*, 2012), we determined the relation between the regression rate of CL c-s area between 13 to 16 and 20 to 23 days and the pregnancy status, considering that the first examination (days 13 to 16 post insemination) is 100%. For more a precise and feasible technique which could be efficiently applied in the field to diagnose pregnancy in cows as early as possible without wasting time by waiting for the next estrus, certain values for the rate of CL c-s area`s change between first and second examinations for pregnancy should be proposed. Inseminated cows can be assertively concluded as non-pregnant if the regression of their CL exceeded 25% from their initial value using ultrasonographic examinations. If the regression is less than 10%, cows can be considered to be most likely conceived, while those which show regression rates between 10-25% are advised to be re-verified on the following day to ensure the accuracy of the regression rate. If the regression rate increases on the second day, then the cows can be considered non-pregnant (Gaja *et al.*, 2009).

Some researchers showed that only less than half of the cows that fail to conceive after first insemination can be detected in estrus according to the expected time following first synchronized insemination (Chenault *et al.*, 2003). Non-pregnant cows that do not return to estrus within the expected time are termed “phantom cows” (Lucy *et al.*, 2004). The existence of phantom cows in the herd poses a serious reproductive challenge. Under the traditional reproductive management, a phantom cow cannot be detected until the pregnancy examination which is usually performed between 40 to 60 days after initial insemination.

Results of our present study using GnRH-TAI protocol proved to be highly successful in overcoming this nagging problem, since the pregnancy rate did not differ between the two groups after the second insemination [100% of GnRH-AI cows (17/17) in group 1 and 81% (13/16) in the Estrus-AI cows in group 2 which exhibited estrus were artificially inseminated]. The pregnancy rates were 35% and 38% in group 1 and 2, respectively.

This result could be due to the injection time of GnRH. Therefore, further investigation is required to decipher the relationship between the day of GnRH injection in relation to the diameter of dominant follicle and the conception rate.

This field study was conducted in the Togo farm of Sendai city, Japan, where the protocol for pregnancy diagnosis is based on rectal palpation on days 45 to 50 post insemination.

The efficacy and importance of field application of our new protocol, a combination of EPD and re-insemination, were clearly evident in this study. For example, it was obvious that this novel combination significantly improved the total pregnancy rate [60 cows out of 81 (74%)] in comparison to the control cows [82/153, 54%] (*P*<0.01). Another benefit that could be gleaned from this study, through focusing on the results of EPD, is that our protocol did not have any detrimental effects such as embryonic mortality. This can be inferred from our results which showed a pregnancy rate of 59% (48/81) in the treatment group after first insemination, when compared to the pregnancy rate of the negative control group of 54% (82/153), which was not statistically different.

In conclusion, our field study demonstrated that re-insemination of non-pregnant cows following EPD is highly efficacious in improving the overall fertility via reducing the inter-insemination and inter-calving intervals. An additional advantage of our protocol is that it assists in early detection of ovarian disorders.
